# Prophylactic Use of Uterine Artery Embolization to Decrease Bleeding in Laparoscopic Myomectomy: A Case Report

**DOI:** 10.7759/cureus.52988

**Published:** 2024-01-26

**Authors:** Swarnima Lunge Patil, Apoorva Dave, Kamlesh Chaudhari

**Affiliations:** 1 Obstetrics and Gynecology, Jawaharlal Nehru Medical College, Datta Meghe Institute of Higher Education and Research, Wardha, IND

**Keywords:** interventional radiologists, hysterectomy, leiomyoma, laparoscopic myomectomy, uterine artery embolization

## Abstract

Uterine fibroids, or leiomyomas, are the most frequent benign tumors affecting the female reproductive system, particularly during the reproductive years. The case report that follows presents the diagnosis and treatment of uterine fibroids in a female patient. The 33-year-old female patient in this instance arrived at the tertiary rural hospital with an abnormally large, bloated belly. Upon examination and imaging, it was discovered that the patient had multiple fibroids growing inside her uterus.

Here, we present a successful management of uterine leiomyoma with laparoscopic myomectomy where we performed uterine artery embolization before surgical management in order to minimize blood loss during surgery. The case highlights the significance of collaboration between gynecologists, surgeons, and interventional radiologists. Thanks to their combined expertise, the patient was given a variety of treatment options, such as minimally invasive treatments, surgical interventions, and medication therapy. Decision considerations included the consequences of fibroids and the patient's age and desire to preserve fertility.

The effect of fibroids on her life expectancy is taken into account. This case emphasizes how important it is to embolize the uterine arteries before having a myomectomy to cure large uterine leiomyomas successfully.

## Introduction

Women between the ages of 14 and 45 have a higher incidence of leiomyoma, a common benign tumor, often known as uterine fibroids. For women of that age, the frequency is nearly as high as 20-40%. These tumors are well-recognized for being infamously asymptomatic and for frequently being discovered by accident during imaging or clinical investigations. Symptoms of leiomyoma include irregular or excessive menstruation, urgent or frequent urination, constipation, dyspareunia, lower back pain, elevated pelvic pressure, urine retention, and lower back discomfort [1].

Ultrasonography is the first chosen imaging modality. Numerous studies examining myomectomy's impact on presenting symptoms have provided valuable insights into the relationship between fibroids and symptoms. Although uterine fibroids are prevalent in women of reproductive age, it was somewhat unexpected to see one in a tertiary clinical setting. There are various treatment modalities for uterine fibroid management such as medical management, myomectomy, hysterectomy, and uterine artery embolization (UAE) [2].

UAE before myomectomy is a recent approach to minimize blood loss during surgery. This case report covers the initial presentation, diagnostic issues, and multidisciplinary therapy approach used in a rural tertiary care hospital for a patient with a large uterine fibroid [3].

This specific case illustrates the clinical subtleties of multiple fibroids and the value of creative management strategies in resource-constrained healthcare environments. We present the clinical data, radiological findings, surgical technique, and postoperative care tailored to the specific requirements of the patient in this report. The example under discussion proves the value of adaptable healthcare approaches in treating complex gynecological problems, particularly in rural locations where access to state-of-the-art medical facilities may be limited.

## Case presentation

A 33-year-old multiparous woman, para 2, live births, both delivered through lower-segment Caesarean section, presented at the rural tertiary care center with complaints of heavy menstrual bleeding and pain in the abdomen for the past two months and associated with white vaginal discharge and lower back pain. On bimanual examination, her abdomen was distended, measuring approximately 22 weeks of gravid uterus.

On palpation, the uterus was firm to hard in consistency with an approximate size of a 22-week gravid uterus and freely mobile with no tenderness. As per vaginal examination, the uterus was 22 weeks in size, freely mobile, firm in consistency, and non-tender, and bilateral fornices were clear.

She had a history of bilateral tubal ligation at the time of the second delivery. She is also a known case of hypothyroidism for the past eight years and currently taking the required medications.

**Figure 1:**
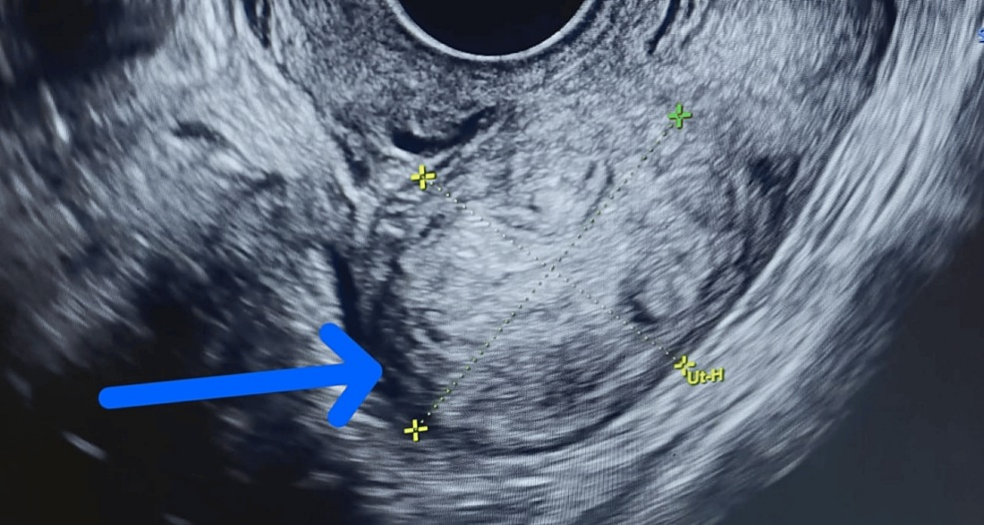
Ultrasonographic image of the suspected uterine fibroid mass

A further ultrasonographic examination showed the presence of multiple uterine fibroids, of which the largest one measured roughly 10×8 cm (depicted in Figure 1).

Chronic menorrhagia caused a considerable decline in hemoglobin levels. The lab investigations of the patient are depicted in Table 1.

**Table 1:** Laboratory parameters preoperatively CRP: C-reactive protein; mL: millilitre; g/dL: gram per decilitre; mg/dL: milligram per decilitre; mEq/L: milliequivalents per litre; mmol/L: millimoles per litre; µL: microlitre; K^+^: potassium; Na^+^: sodium

Serum parameters	Levels recorded	Normal range
White blood cell	11.2×10^5^/mL	4-11×10^5^/mL
Hemoglobin	8.4 gm/dL	12-17 g/dL
CRP	7 mg/dL	0.3-1 mg/dL
Na^+^	138 mEq/L	135-145 mEq/L
K^+^	4 mmol/L	3.5-5.5 mmol/L
Lactate	1.6 mmol/L	0.5-2 mmol/L
Platelet	4.23×10^5^/µL	1.5-3×10^5^/µL

The patient was informed about all the available medical and surgical treatment options. Given the large fibroid and related symptoms, she decided to proceed with UAE followed by a laparoscopic myomectomy. The patient was taken for UAE before the surgery to minimize blood loss during surgery since the woman was already anemic.

To prevent blood loss during myomectomy, laparoscopy was preceded by UAE. Aseptic procedures were followed during the UAE, and a 5F sheath was used to get access to the right femoral artery. Using a 5F cobra catheter, the bilateral iliac artery was cannulated. The bilateral uterine arteries were then selectively cannulated using Endocryl glue. A post-embolization angiogram was performed to ensure successful devascularization. Embolization was accomplished in the interventional radiology department. The patient tolerated the procedure well, and her vitals were stable.

**Figure 2:**
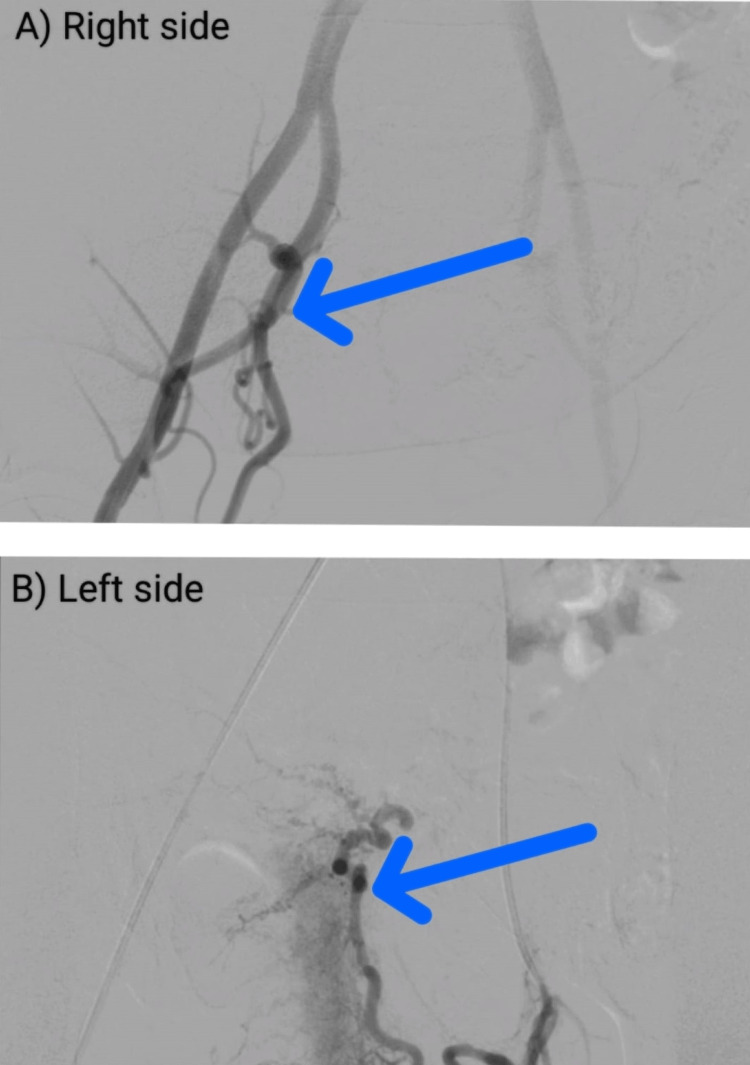
An X-ray image of the angiogram done after the uterine artery embolization: A) right side and B) left side

Given below is an X-ray image of the angiogram done after the UAE (Figure 2).

Intraoperative steps and findings

Following the patient's valid consent, general anesthesia was administered, the patient was placed in the desired posture, and painting, cleaning, and draping of parts were completed. A 5-mm port was inserted near the location of Palmer’s point. Then pneumoperitoneum was created, and a 10-mm paraumbilical port and two more 5-mm ports were introduced.

A myoma measuring 10×9 cm was discovered on the posterior wall of the uterus on laparoscopy. It was injected with 20 units of vasopressin into 300 millilitres of normal saline, followed by a transverse incision made on the most visible surface of the posterior wall of the uterus using a harmonic scalpel. A myoma screw helped to hold the myoma in place, and the traction and countertraction approach was used to remove the myoma.

**Figure 3:**
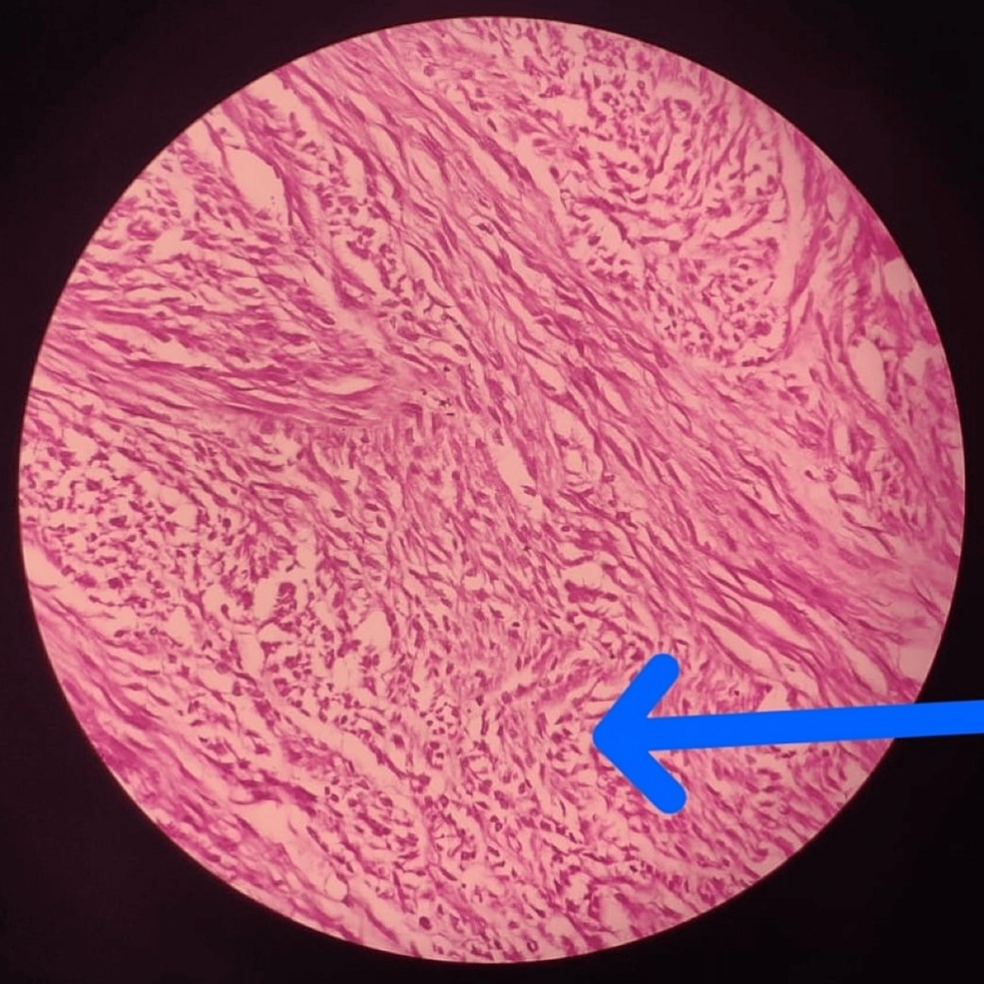
Histopathological image of the resected uterine fibroid showing bundles of spindle-shaped smooth muscle cells and a whorled or swirling pattern of growth

The fibrous strands that were linked to the myoma bed were cut off, and the myoma was separated from the myoma bed using a harmonic scalpel. The myoma was morcellated and removed, and hemostasis was satisfactory. There was no open endometrial cavity. The barbed suture was used to sew the uterine musculature and the myoma bed. Each port was removed under direct vision. Vicryl 2-0 was used for port closure. The intraoperative period was uneventful. There was no evident blood loss. The specimen was sent for histopathological examination, which revealed a leiomyoma as suspected. The slide showed bundles of spindle-shaped smooth muscle cells and a whorled or swirling pattern of growth as shown in the figure below (Figure 3).

A cut portion of the fibroid was inspected to look for potential lesions. Upon gross examination, the mass showed no evidence of hemorrhagic or necrotic tissues and had a pink color. It had a uniform texture and was well-circumscribed.

Around 10 ml of blood was lost during surgery, which is acceptable considering the woman's anemia. The surgery recuperation process proceeded smoothly for the patient. She was discharged from the hospital with instructions for follow-up care on the fifth postoperative day. Her hemoglobin levels improved gradually, and she experienced an improvement in her symptoms from the next cycle onwards.

## Discussion

Uterine fibroids affect 20-40% of women of reproductive age. Factors such as advanced age, early menarche, obesity, nulliparity, and family history can raise the risk of fibroids. In India, 17 out of 1000 married women get a hysterectomy each year due to fibroids, according to a 2018 survey. Benign tumors called fibroids arise from the uterine smooth muscle tissue or myometrium. Fibroid formation requires the hormones progesterone and estrogen [4,5].

Fibroids are rare before puberty, more prevalent throughout reproductive times, and less common after menopause. Aromatase in fibroid tissue produces estrogen endogenously, and progesterone and estrogen receptors are expressed by fibroid stem cells. The presence of these hormones stimulates tumor growth [6]. Uterine fibroids are categorized as intramural, which is located inside the myometrium, subserosal, which is located outside the uterus, and submucosal, which is located inside the uterine cavity. Fibroid symptoms and therapy are influenced by the fibroids' number, size, and location [7]. Similarly, in the patient in question, myomectomy and hysterectomy were the only alternatives for therapy due to the large size of the fibroid.

Ultrasonography is the first step in a fibroid examination since it provides trustworthy data. Other techniques include transvaginal ultrasonography, which has a slight limitation in that it is unable to identify subserosal fibroids. Nevertheless, it is capable of finding uterine fibroids 90-99% of the time [8].

A well-tried preoperative technique that reduces excessive blood loss after surgery is UAE. Appropriate individuals who meet the following requirements, being a premenopausal woman, having a history of heavy menstruation, and not wanting to get pregnant, are suggested to have this surgery [9]. In this instance, the difficulties involved in managing gigantic uterine fibroids, especially in a rural tertiary care setting, are due in large part to their size and location. This is typically brought on by a lack of awareness and understanding as well as a decline in the number of care facilities in primary healthcare facilities.

When treating fibroids, there are two different treatment options available: surgical and medical. Only a few fibroids can benefit from medical therapy, or in women who are perimenopausal or menopausal, it can be used as a preoperative treatment to shrink the tumor before surgery. Oral contraceptives, tranexamic acid, ulipristal, and the levonorgestrel-releasing intrauterine device (Mirena, Bayer Healthcare Pharmaceuticals, Berlin, Germany) are the medications that are utilized. When fibroid proportions and symptoms represent significant health hazards, as this example illustrates, a laparoscopic myomectomy may be the most effective treatment option [10].

UAE has benefits over the gold standard hysterectomy or myomectomy, including cost-effectiveness, uterine sparing, and a quicker recovery time. Compared to myomectomy, UAE is associated with fewer perioperative complications. Pregnancy, active pelvic inflammatory disorders, and gynecological malignancies are all absolute contraindications to using UAE. Pain is the most typical acute postoperative consequence and lasts for 24-48 hours after the treatment. Non-steroidal anti-inflammatory drugs (NSAIDs), superior hypogastric nerve blockade, morphine or fentanyl infusion, and other techniques have all been utilized to manage this discomfort [11].

Patients can be treated with oral NSAIDs and oral opioids, if necessary, once the acute pain has subsided. Non-purulent vaginal discharge, an infection, a fever, and post-embolization syndrome are other side effects. None of the research group's participants could be evaluated for the potential that the UAE could reduce fertility, which has not yet been proven [12]. The absence of sufficient research to comment on the fertility results post UAE should be thoroughly discussed with young women who seek to become pregnant after treatment. However, numerous studies have demonstrated successful pregnancies after UAE.

A preventive pre-procedure dose of the IV antibiotic ceftriaxone lowered the risk of infection. A long-term consequence linked to UAE is a decline in ovarian reserve, but this risk varies depending on a woman's age, with a higher failure risk in women over 40 (45%) and a lower failure risk in women under 40 (less than 1%). Long-term issues including symptom recurrence and the necessity for further treatment could not be determined.

Using a tourniquet at the level of the isthmus to compress the uterine artery, using a myomectomy clamp, using an injection of vasopressin, and putting the patient under hypotensive anesthesia before surgery are further techniques that can assist in reducing blood loss during a myomectomy. However, out of all of these, we have chosen the UAE since it also has the benefit of shrinking the size of fibroids that cannot be operated upon in the coming months.

Ngeh et al. reported on five patients who received preoperative embolization to lower/avoid the danger of hysterectomy and lessen the possibility of serious blood loss [13]. All patients had comparatively little blood loss, and none of them later needed blood transfusions. This stood in stark contrast to the outcomes that were disclosed for the control group.

Retrospective findings of 21 patients who had preoperative UAE before myomectomy were published in 2008 by Tixier et al. The surgeons reported that myoma excision resulted in very little blood loss and that the intraoperative suturing of the uterus was technically easier. Minimal blood loss and an easy surgical procedure were also seen in our study [14].

A different French group, Butori et al., published a report in 2011 on a retrospective analysis of 33 women with at least one myoma (mean diameter 9 cm (5-15 cm)) who were between the ages of 24 and 45. Surgeons reported that myoma removal was easier while doing laparotomy or laparoscopic myomectomy procedures [15]. There were no appreciable variations in the preoperative and postoperative hemoglobin levels, and no patient needed a blood transfusion. Additionally, our patient did not require a blood transfusion.

In our case, only the large visible myoma was excised and small myomas were not removed laparoscopically because UAE would regress them eventually and help in managing the symptoms associated with myomas.

## Conclusions

The article does a good job of highlighting the patient's successful care as well as the value of a customized, multidisciplinary strategy that includes an interventional radiologist and an anesthetist to meet the patient's unique needs. In this instance, a 33-year-old lady with multiple uterine fibroids experienced pelvic pain, pressure feelings, and heavy menstruation. Imaging studies confirmed the diagnosis, which aided in the planning and consideration of possible treatments. The significance of tailored treatment and collaborative decision-making in the handling of uterine fibroids is emphasized by this case study. Through careful consideration of the patient's needs and a well-timed recovery, a laparoscopic myomectomy preceded by UAE was performed, leading to the patient's enhanced quality of life and symptom relief.
